# Bis{deca­carbonyl­bis­[μ-2,2′-(phenyl­imino)­diethano­lato]ditin(II)ditungsten(0)(2 *Sn—W*)} hexa­carbonyl­tungsten(0)

**DOI:** 10.1107/S1600536810019343

**Published:** 2010-05-29

**Authors:** Thorsten Berends, Ljuba Iovkova, Edward R. T. Tiekink, Klaus Jurkschat

**Affiliations:** a Fakultät Chemie, Technische Universität Dortmund, 44221 Dortmund, Germany; b Department of Chemistry, University of Malaya, 50603 Kuala Lumpur, Malaysia

## Abstract

In the title 2:1 adduct, [Sn_2_W_2_(C_10_H_13_NO_2_)_2_(CO)_10_]_2_[W(CO)_6_], the complete hexa­carbonyl­tungsten mol­ecule is generated by a crystallographic inversion centre. The heterometallic mol­ecule features a central Sn_2_O_2_ core with essentially equal Sn—O_eth­oxy_ bond lengths. The second eth­oxy O and amine N atoms of each *N*,*O*,*O*′-tridentate ligand coordinate to one Sn atom only. The NO_3_ donor atoms occupy basal positions and the W atom the apical position in a distorted square-pyramidal geometry for each Sn atom. The W atoms are approximately *syn* to each other but the central metal core is non-planar [W—Sn⋯Sn—W pseudo-torsion angle = 43.573 (16)°]. One of the carbonyl ligands in the heterometallic mol­ecule is disordered over two orientations with equal occupancies. In the crystal, the heterometallic mol­ecules associate *via* C—H⋯O inter­actions, forming supra­molecular layers with undulating topology in the *ab* plane. These stack along the *c* axis, defining voids which are occupied by the W(CO)_6_ mol­ecules.

## Related literature

For synthetic background, see: Zeldin & Gsell (1976 ▶); Zschunke *et al.* (1983 ▶, 1986 ▶). For related structures, see: Berends *et al.* (2009 ▶). For additional geometric analysis, see: Addison *et al.* (1984 ▶).
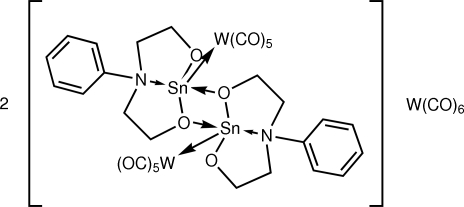


## Experimental

### 

#### Crystal data

[Sn_2_W_2_(C_10_H_13_NO_2_)_2_(CO)_10_]_2_[W(CO)_6_]
*M* *_r_* = 2839.15
Triclinic, 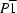

*a* = 11.3547 (5) Å
*b* = 12.5454 (5) Å
*c* = 16.8187 (7) Å
α = 108.715 (4)°
β = 92.758 (4)°
γ = 115.350 (4)°
*V* = 2001.90 (19) Å^3^
*Z* = 1
Mo *K*α radiation
μ = 8.46 mm^−1^
*T* = 173 K
0.20 × 0.08 × 0.06 mm

#### Data collection

Oxford Diffraction Xcalibur2 CCD diffractometer
Absorption correction: multi-scan (*CrysAlis RED*; Oxford Diffraction, 2008 ▶) *T*_min_ = 0.608, *T*_max_ = 1.000
39646 measured reflections
9035 independent reflections
7218 reflections with *I* > 2σ(*I*)
*R*_int_ = 0.038

#### Refinement

*R*[*F* ^2^ > 2σ(*F* ^2^)] = 0.020
*wR*(*F* ^2^) = 0.039
*S* = 0.95
9035 reflections
518 parameters
12 restraints
H-atom parameters constrained
Δρ_max_ = 0.87 e Å^−3^
Δρ_min_ = −1.74 e Å^−3^

### 

Data collection: *CrysAlis CCD* (Oxford Diffraction, 2008 ▶); cell refinement: *CrysAlis RED* (Oxford Diffraction, 2008 ▶); data reduction: *CrysAlis RED*; program(s) used to solve structure: *SHELXS97* (Sheldrick, 2008 ▶); program(s) used to refine structure: *SHELXL97* (Sheldrick, 2008 ▶); molecular graphics: *ORTEP-3* (Farrugia, 1997 ▶) and *DIAMOND* (Brandenburg, 2006 ▶); software used to prepare material for publication: *publCIF* (Westrip, 2010 ▶).

## Supplementary Material

Crystal structure: contains datablocks global, I. DOI: 10.1107/S1600536810019343/hb5458sup1.cif

Structure factors: contains datablocks I. DOI: 10.1107/S1600536810019343/hb5458Isup2.hkl

Additional supplementary materials:  crystallographic information; 3D view; checkCIF report

## Figures and Tables

**Table 1:** Selected bond lengths (Å)

Sn1—W1	2.7274 (3)
Sn1—O11	2.091 (2)
Sn1—O17	2.001 (3)
Sn1—O21	2.201 (2)
Sn1—N14	2.507 (3)
Sn2—W2	2.7334 (3)
Sn2—O11	2.173 (2)
Sn2—O21	2.104 (2)
Sn2—O27	2.011 (2)
Sn2—N24	2.391 (3)

**Table 2:** Hydrogen-bond geometry (Å, °)

*D*—H⋯*A*	*D*—H	H⋯*A*	*D*⋯*A*	*D*—H⋯*A*
C23—H23a⋯O44^i^	0.99	2.52	3.301 (5)	136
C64—H64a⋯O31^ii^	0.95	2.58	3.174 (6)	121

Symmetry codes: (i) 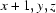; (ii) 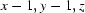.

**Fig. 1.**
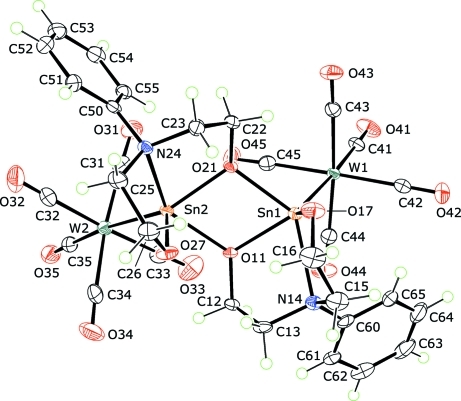
The molecular structure of [(CO)5WSn(OC2H4)2NPh]2 in (I) showing displacement ellipsoids at the 50% probability level. One one orientation of the disordered carbonyl group (bound to W2) is shown.

**Fig. 2.**
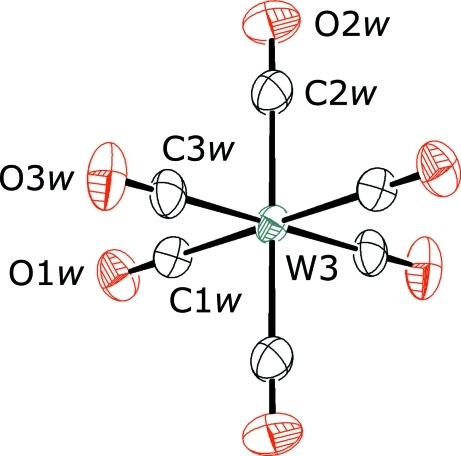
The molecular structure of the W(CO)6 molecule in (I) showing displacement ellipsoids at the 50% probability level. Unlabelled atoms are related by the symmetry operation -x, -y, -z.

**Fig. 3.**
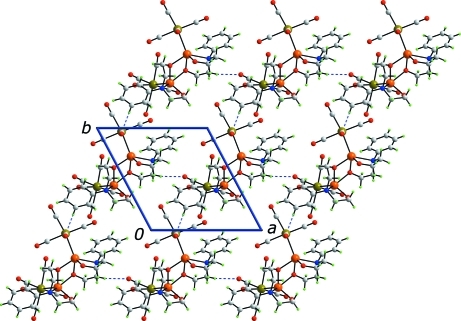
Two-dimensional array, in the ab plane, in (I) mediated by C–H···O interactions (blue dashed lines) between molecules of [(CO)5WSn(OC2H4)2NPh]2. Colour code: W, olive; Sn, orange; O, red; N, blue; C, grey; and H, green.

**Fig. 4.**
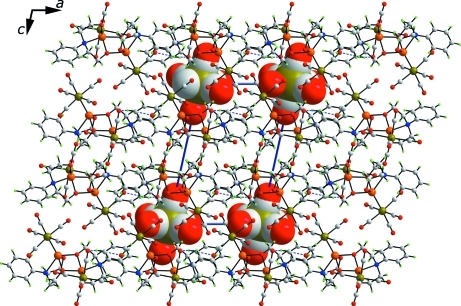
A view of the stacking of layers in (I) along the c axis allowing for the incorporation of W(CO)6 molecules (shown in space filling mode) in the voids thus formed. The C–H···O interactions are shown as blue dashed lines. Colour code: W, olive; Sn, orange; O, red; N, blue; C, grey; and H, green.

## supplementary crystallographic information

### Comment

The title compound, (I), investigated as a continuation of a long-standing interest in heterometallic compounds containing Sn and W (Zeldin *et al.*, 1976; Zschunke *et al.*, 1983; Zschunke *et al.*, 1986; Berends *et al.*, 2009), is a co-crystal comprising a [(CO)_5_WSn(OC_2_H_4_)_2_NPh]_2_ molecule, Fig. 1, and a W(CO)_6_ molecule, Fig. 2, with the latter disposed about a crystallographic centre of inversion.

The structure of [(CO)_5_WSn(OC_2_H_4_)_2_NPh]_2_ resembles that of the related *N-*methyl- and *N*-(*t*-butyl)- substituted derivatives (Berends *et al.*, 2009). Dimerization is achieved by almost symmetric µ_2_-ethoxy bridges between the Sn atoms, Table 1. Each tridentate ligand also coordinates to a tin atom via bonds formed by a second ethoxy-O and the amine-N. The coordination geometries are based on square pyramidal configurations with the O11, O17, O21, and N14 atoms at Sn1, and the O11, O21, O27, and N24 atoms at Sn2 occupying the basal positions, and W1 (at Sn1) and W2 (at Sn2) occupying the apical positions, Table 1. The values of τ = 0.02 and 0.08 for Sn1 and Sn2, respectively, which compare to τ = 0.0 for an ideal square pyramid and τ = 1.0 for an ideal trigonal pyramidal arrangement (Addison *et al.*, 1984), confirm the assignment of coordination polyhedra. The W atoms are approximately *syn* to each other but the W1–Sn1···Sn2–W2 atoms deviate from co-planarity as seen in the torsion angle of 43.573 (16) °. The Sn1–N14 and Sn2–N24 bond distances of 2.507 (3) and 2.391 (3) Å, respectively, fall in between those found for the *N*-methyl (2.356 (5)/2.360 (6) Å) and *N*-(*t*-butyl) (2.549 (4)/2.444 (5) Å)) -substituted analogues (Berends *et al.*, 2009) and indicate the increasing donor capacity of the N atoms in the sequence N(*t*-Bu) < NPh < NMe.

The most prominent intermolecular interactions operating in the crystal structure are of the type C–H···O, Table 2, and these occur between atoms comprising [(CO)_5_WSn(OC_2_H_4_)_2_NPh]_2_ to form an undulating 2-D array in the *ab* plane, Fig. 3. Centrosymmetric layers associate to form a double layer and these stack along the *c* axis. Gaps evident in Fig. 2, from translational symmetry, face each other in the global crystal packing to form voids of approximate volume 300 Å^3^, allowing for the incorporation of the W(CO)_6_ molecules, as highlighted in Fig. 4.

### Experimental

Freshly prepared tin(II) butoxide was reacted with one molar equivalent of *N*-phenyldiethanolamine in toluene. The toluene/butanol azeotropic mixture was distilled off and the stannylene Sn(OCH_2_CH_2_)_2_NPh was isolated as a very poorly soluble white solid that was not characterized further and used in the next step without further purification. The stannylene (2.9 g, 4.8 mmol) was suspended in THF (50 ml) and an excess of W(CO)_5_.THF in THF was added dropwise. The reaction mixture was stirred for 24 h at room temperature during which it turned to a clear solution. The THF was removed *in vacuo* and the residue was recrystallized from toluene to give colourless prisms of (I) (5.2 g, 75%, m.p. 460 K). ^119^Sn-NMR (CD_2_Cl_2_, 300 MHz) δ -210 p.p.m. (s, ^1^*J*(^119^Sn-^183^W) = 1515 Hz). Elemental analysis: calculated (%) for C_66_H_52_N_4_O_34_Sn_4_W_5_: C 27.9, H 1.9, N 2.0. Found: C 27.0, H 2.0, N 1.9.

### Refinement

The H atoms were geometrically placed (C—H = 0.95–0.99 Å) and refined as riding with *U*_iso_(H) = 1.2*U*_eq_(C). The maximum and minimum residual electron density peaks of 0.87 and -1.74 e Å^-3^, respectively, were located 0.00 and 0.57 Å from the W3 atom. One the W2-bound carbonyl groups (C35≡O35) was disordered over two positions of equal weight (from anisotropic refinement). The anisotropic displacement parameters of the disordered atoms were constrained to be equal and approximately isotropic by the EADP and ISOR commands in SHELX-97, respectively (Sheldrick, 2008).

### Crystal data

**Table:**

[Sn_2_W_2_(C_10_H_13_NO_2_)_2_(CO)_10_]_2_[W(CO)_6_]	*Z* = 1
*M**_r_* = 2839.15	*F*(000) = 1318
Triclinic, *P*1	*D*_x_ = 2.355 Mg m^−^^3^
Hall symbol: -P 1	Melting point: 460 K
*a* = 11.3547 (5) Å	Mo *K*α radiation, λ = 0.71073 Å
*b* = 12.5454 (5) Å	Cell parameters from 35730 reflections
*c* = 16.8187 (7) Å	θ = 2.0–25.5°
α = 108.715 (4)°	µ = 8.46 mm^−^^1^
β = 92.758 (4)°	*T* = 173 K
γ = 115.350 (4)°	Prism, colourless
*V* = 2001.90 (19) Å^3^	0.20 × 0.08 × 0.06 mm

### Data collection

**Table:**

Oxford Diffraction Xcalibur2 CCD diffractometer	9035 independent reflections
Radiation source: Enhance (Mo) X-ray Source	7218 reflections with *I* > 2σ(*I*)
graphite	*R*_int_ = 0.038
Detector resolution: 16.0560 pixels mm^-1^	θ_max_ = 27.5°, θ_min_ = 2.0°
973 frames via ω–rotation (Δω = 1°) and two times 30 s per frame (16 sets at different κ–angles) scans	*h* = −14→14
Absorption correction: multi-scan (*CrysAlis RED*; Oxford Diffraction, 2008)	*k* = −16→15
*T*_min_ = 0.608, *T*_max_ = 1.000	*l* = −21→21
39646 measured reflections	

### Refinement

**Table:**

Refinement on *F*^2^	Primary atom site location: structure-invariant direct methods
Least-squares matrix: full	Secondary atom site location: difference Fourier map
*R*[*F*^2^ > 2σ(*F*^2^)] = 0.020	Hydrogen site location: inferred from neighbouring sites
*wR*(*F*^2^) = 0.039	H-atom parameters constrained
*S* = 0.95	*w* = 1/[σ^2^(*F*_o_^2^) + (0.018*P*)^2^] where *P* = (*F*_o_^2^ + 2*F*_c_^2^)/3
9035 reflections	(Δ/σ)_max_ = 0.001
518 parameters	Δρ_max_ = 0.87 e Å^−^^3^
12 restraints	Δρ_min_ = −1.74 e Å^−^^3^

### Special details

**Table:**

Geometry. All s.u.'s (except the s.u. in the dihedral angle between two l.s. planes) are estimated using the full covariance matrix. The cell s.u.'s are taken into account individually in the estimation of s.u.'s in distances, angles and torsion angles; correlations between s.u.'s in cell parameters are only used when they are defined by crystal symmetry. An approximate (isotropic) treatment of cell s.u.'s is used for estimating s.u.'s involving l.s. planes.
Refinement. Refinement of *F*^2^ against ALL reflections. The weighted *R*-factor wR and goodness of fit *S* are based on *F*^2^, conventional *R*-factors *R* are based on *F*, with *F* set to zero for negative *F*^2^. The threshold expression of *F*^2^ > 2σ(*F*^2^) is used only for calculating *R*-factors(gt) etc. and is not relevant to the choice of reflections for refinement. *R*-factors based on *F*^2^ are statistically about twice as large as those based on *F*, and *R*- factors based on ALL data will be even larger.

### Fractional atomic coordinates and isotropic or equivalent isotropic displacement parameters (Å^2^)

**Table:**

	*x*	*y*	*z*	*U*_iso_*/*U*_eq_	Occ. (<1)
W1	−0.282538 (14)	0.425012 (14)	0.091913 (8)	0.01414 (4)	
W2	0.204122 (15)	0.964916 (14)	0.386429 (9)	0.02111 (4)	
W3	0.0000	0.0000	0.0000	0.02888 (6)	
Sn1	−0.12400 (2)	0.43967 (2)	0.226266 (13)	0.01384 (5)	
Sn2	0.17113 (2)	0.72211 (2)	0.335720 (13)	0.01364 (5)	
O11	−0.0388 (2)	0.6118 (2)	0.33287 (13)	0.0172 (5)	
O17	−0.0577 (3)	0.3220 (2)	0.24211 (15)	0.0270 (6)	
O21	0.0794 (2)	0.5636 (2)	0.21882 (13)	0.0159 (5)	
O27	0.2066 (2)	0.6510 (2)	0.42020 (14)	0.0225 (6)	
O31	0.1889 (3)	0.9430 (3)	0.19202 (17)	0.0344 (7)	
O32	0.5203 (3)	1.0749 (3)	0.4149 (2)	0.0577 (10)	
O33	−0.1126 (3)	0.8466 (3)	0.3463 (2)	0.0481 (8)	
O34	0.1939 (3)	0.9427 (3)	0.56971 (17)	0.0458 (9)	
O41	−0.4503 (3)	0.4212 (3)	−0.06435 (16)	0.0363 (7)	
O42	−0.4920 (3)	0.1343 (3)	0.04587 (17)	0.0441 (8)	
O43	−0.0948 (3)	0.3502 (3)	−0.02380 (16)	0.0328 (7)	
O44	−0.4392 (3)	0.5231 (3)	0.22452 (17)	0.0350 (7)	
O45	−0.0934 (3)	0.7209 (3)	0.13766 (19)	0.0456 (8)	
O1W	−0.2172 (3)	0.0976 (3)	0.0011 (2)	0.0499 (8)	
O2W	0.2380 (4)	0.2807 (3)	0.0516 (2)	0.0713 (12)	
O3W	−0.0041 (3)	0.0494 (4)	0.1977 (2)	0.0575 (10)	
N14	−0.2180 (3)	0.3899 (3)	0.34934 (17)	0.0173 (6)	
N24	0.3492 (3)	0.6808 (3)	0.29092 (16)	0.0150 (6)	
C12	−0.0998 (4)	0.6237 (3)	0.4054 (2)	0.0210 (8)	
H12A	−0.1826	0.6285	0.3915	0.025*	
H12B	−0.0382	0.7023	0.4549	0.025*	
C13	−0.1313 (3)	0.5090 (4)	0.4276 (2)	0.0208 (8)	
H13A	−0.1787	0.5120	0.4755	0.025*	
H13B	−0.0476	0.5084	0.4462	0.025*	
C15	−0.1904 (4)	0.2831 (4)	0.3487 (2)	0.0275 (9)	
H15A	−0.2646	0.2009	0.3089	0.033*	
H15B	−0.1842	0.2809	0.4070	0.033*	
C16	−0.0601 (4)	0.3013 (4)	0.3201 (2)	0.0304 (9)	
H16A	0.0156	0.3752	0.3655	0.037*	
H16B	−0.0490	0.2248	0.3124	0.037*	
C22	0.1591 (3)	0.5150 (3)	0.1702 (2)	0.0187 (8)	
H22A	0.1856	0.5545	0.1273	0.022*	
H22B	0.1058	0.4217	0.1391	0.022*	
C23	0.2809 (3)	0.5439 (3)	0.2290 (2)	0.0195 (8)	
H23A	0.3436	0.5269	0.1945	0.023*	
H23B	0.2555	0.4873	0.2617	0.023*	
C25	0.4201 (4)	0.6963 (4)	0.3742 (2)	0.0229 (8)	
H25A	0.4738	0.7881	0.4102	0.027*	
H25B	0.4814	0.6587	0.3632	0.027*	
C26	0.3187 (4)	0.6303 (4)	0.4220 (2)	0.0242 (9)	
H26A	0.2883	0.5375	0.3961	0.029*	
H26B	0.3639	0.6612	0.4827	0.029*	
C31	0.1962 (4)	0.9539 (4)	0.2626 (2)	0.0242 (8)	
C32	0.4076 (4)	1.0356 (4)	0.4052 (2)	0.0343 (10)	
C33	0.0011 (4)	0.8895 (4)	0.3611 (2)	0.0291 (9)	
C34	0.2003 (4)	0.9537 (4)	0.5052 (2)	0.0305 (10)	
C35	0.2013 (8)	1.1351 (9)	0.4227 (5)	0.0224 (13)	0.50
O35	0.2001 (6)	1.2300 (6)	0.4454 (4)	0.0326 (11)	0.50
C35'	0.2389 (9)	1.1409 (9)	0.4413 (5)	0.0224 (13)	0.50
O35'	0.2527 (6)	1.2454 (6)	0.4726 (4)	0.0326 (11)	0.50
C41	−0.3904 (4)	0.4216 (3)	−0.0074 (2)	0.0206 (8)	
C42	−0.4151 (4)	0.2378 (4)	0.0629 (2)	0.0246 (9)	
C43	−0.1640 (4)	0.3754 (3)	0.0168 (2)	0.0197 (8)	
C44	−0.3857 (4)	0.4845 (4)	0.1755 (2)	0.0212 (8)	
C45	−0.1578 (4)	0.6146 (4)	0.1211 (2)	0.0249 (9)	
C50	0.4344 (3)	0.7664 (3)	0.25168 (19)	0.0164 (7)	
C51	0.5718 (3)	0.8100 (4)	0.2657 (2)	0.0225 (8)	
H51A	0.6129	0.7849	0.3016	0.027*	
C52	0.6483 (4)	0.8903 (4)	0.2268 (2)	0.0304 (10)	
H52A	0.7422	0.9209	0.2370	0.037*	
C53	0.5899 (4)	0.9265 (4)	0.1735 (2)	0.0305 (10)	
H53A	0.6432	0.9809	0.1467	0.037*	
C54	0.4533 (4)	0.8831 (4)	0.1593 (2)	0.0255 (9)	
H54A	0.4124	0.9080	0.1231	0.031*	
C55	0.3757 (4)	0.8024 (3)	0.1985 (2)	0.0186 (8)	
H55A	0.2819	0.7720	0.1884	0.022*	
C60	−0.3583 (4)	0.3562 (3)	0.3345 (2)	0.0204 (8)	
C61	−0.4104 (4)	0.4260 (4)	0.3887 (2)	0.0210 (8)	
H61A	−0.3540	0.4971	0.4394	0.025*	
C62	−0.5461 (4)	0.3915 (4)	0.3686 (3)	0.0318 (10)	
H62A	−0.5816	0.4382	0.4067	0.038*	
C63	−0.6283 (4)	0.2915 (4)	0.2949 (3)	0.0378 (11)	
H63A	−0.7201	0.2693	0.2814	0.045*	
C64	−0.5764 (4)	0.2235 (4)	0.2402 (3)	0.0361 (11)	
H64A	−0.6326	0.1548	0.1885	0.043*	
C65	−0.4425 (4)	0.2545 (4)	0.2601 (2)	0.0253 (9)	
H65A	−0.4085	0.2056	0.2225	0.030*	
C1W	−0.1393 (4)	0.0640 (4)	−0.0008 (3)	0.0347 (10)	
C3W	−0.0019 (4)	0.0314 (5)	0.1270 (3)	0.0398 (11)	
C2W	0.1544 (5)	0.1819 (5)	0.0329 (3)	0.0454 (12)	

### Atomic displacement parameters (Å^2^)

**Table:**

	*U*^11^	*U*^22^	*U*^33^	*U*^12^	*U*^13^	*U*^23^
W1	0.01393 (7)	0.01671 (8)	0.01116 (7)	0.00708 (6)	0.00274 (5)	0.00467 (6)
W2	0.02669 (9)	0.01450 (8)	0.02054 (8)	0.00916 (7)	0.01048 (6)	0.00461 (6)
W3	0.03388 (14)	0.02716 (13)	0.03009 (12)	0.01872 (11)	0.00676 (10)	0.01009 (10)
Sn1	0.01483 (12)	0.01582 (12)	0.01151 (11)	0.00799 (10)	0.00312 (9)	0.00475 (9)
Sn2	0.01529 (12)	0.01461 (12)	0.01094 (11)	0.00749 (10)	0.00403 (9)	0.00392 (9)
O11	0.0165 (13)	0.0191 (13)	0.0132 (11)	0.0072 (11)	0.0085 (9)	0.0037 (10)
O17	0.0395 (17)	0.0323 (16)	0.0247 (13)	0.0257 (14)	0.0150 (12)	0.0159 (12)
O21	0.0124 (12)	0.0189 (13)	0.0130 (11)	0.0078 (11)	0.0049 (9)	0.0009 (10)
O27	0.0251 (14)	0.0355 (16)	0.0200 (13)	0.0190 (13)	0.0118 (11)	0.0185 (12)
O31	0.0388 (18)	0.0384 (18)	0.0331 (16)	0.0190 (15)	0.0119 (13)	0.0203 (14)
O32	0.0267 (19)	0.050 (2)	0.055 (2)	0.0002 (17)	0.0024 (15)	−0.0043 (17)
O33	0.037 (2)	0.055 (2)	0.071 (2)	0.0307 (18)	0.0195 (16)	0.0333 (18)
O34	0.066 (2)	0.045 (2)	0.0193 (15)	0.0225 (18)	0.0110 (14)	0.0081 (14)
O41	0.0443 (18)	0.054 (2)	0.0186 (14)	0.0351 (17)	0.0006 (12)	0.0076 (13)
O42	0.0468 (19)	0.0227 (17)	0.0334 (16)	−0.0042 (15)	0.0089 (14)	0.0038 (13)
O43	0.0352 (17)	0.0425 (18)	0.0271 (14)	0.0231 (15)	0.0172 (13)	0.0123 (13)
O44	0.0442 (18)	0.0503 (19)	0.0340 (16)	0.0359 (16)	0.0223 (14)	0.0229 (14)
O45	0.044 (2)	0.0199 (17)	0.058 (2)	0.0040 (15)	0.0155 (16)	0.0120 (15)
O1W	0.048 (2)	0.059 (2)	0.066 (2)	0.0393 (19)	0.0203 (17)	0.0303 (18)
O2W	0.064 (3)	0.033 (2)	0.090 (3)	0.007 (2)	0.015 (2)	0.012 (2)
O3W	0.079 (3)	0.087 (3)	0.0382 (19)	0.063 (2)	0.0235 (18)	0.0261 (19)
N14	0.0214 (16)	0.0181 (16)	0.0163 (14)	0.0112 (14)	0.0057 (12)	0.0085 (12)
N24	0.0151 (15)	0.0188 (16)	0.0112 (13)	0.0085 (13)	0.0019 (11)	0.0050 (12)
C12	0.0188 (19)	0.023 (2)	0.0134 (17)	0.0068 (17)	0.0074 (14)	0.0014 (15)
C13	0.0186 (19)	0.035 (2)	0.0126 (17)	0.0128 (18)	0.0056 (14)	0.0134 (16)
C15	0.043 (3)	0.027 (2)	0.027 (2)	0.023 (2)	0.0124 (18)	0.0172 (17)
C16	0.043 (3)	0.037 (2)	0.029 (2)	0.030 (2)	0.0087 (18)	0.0186 (19)
C22	0.0184 (19)	0.0187 (19)	0.0151 (17)	0.0086 (16)	0.0062 (14)	0.0016 (15)
C23	0.0187 (19)	0.0175 (19)	0.0213 (18)	0.0088 (16)	0.0055 (15)	0.0053 (15)
C25	0.023 (2)	0.032 (2)	0.0154 (17)	0.0136 (18)	−0.0002 (15)	0.0104 (16)
C26	0.029 (2)	0.030 (2)	0.0206 (19)	0.0160 (19)	0.0043 (16)	0.0146 (17)
C31	0.026 (2)	0.020 (2)	0.033 (2)	0.0135 (18)	0.0109 (17)	0.0136 (17)
C32	0.036 (3)	0.025 (2)	0.024 (2)	0.005 (2)	0.0062 (18)	−0.0006 (18)
C33	0.035 (3)	0.030 (2)	0.038 (2)	0.025 (2)	0.0163 (19)	0.0186 (19)
C34	0.030 (2)	0.019 (2)	0.027 (2)	0.0052 (18)	0.0046 (17)	−0.0022 (17)
C35	0.0225 (16)	0.0222 (15)	0.0222 (15)	0.0103 (10)	0.0059 (11)	0.0082 (9)
O35	0.0336 (14)	0.0310 (12)	0.0332 (13)	0.0160 (9)	0.0099 (10)	0.0107 (9)
C35'	0.0225 (16)	0.0222 (15)	0.0222 (15)	0.0103 (10)	0.0059 (11)	0.0082 (9)
O35'	0.0336 (14)	0.0310 (12)	0.0332 (13)	0.0160 (9)	0.0099 (10)	0.0107 (9)
C41	0.023 (2)	0.024 (2)	0.0140 (17)	0.0125 (17)	0.0034 (15)	0.0035 (15)
C42	0.028 (2)	0.025 (2)	0.0149 (18)	0.0094 (19)	0.0060 (16)	0.0043 (16)
C43	0.024 (2)	0.0195 (19)	0.0161 (17)	0.0101 (17)	0.0034 (15)	0.0079 (15)
C44	0.022 (2)	0.024 (2)	0.0182 (18)	0.0097 (18)	0.0008 (15)	0.0120 (16)
C45	0.023 (2)	0.027 (2)	0.024 (2)	0.0123 (19)	0.0089 (16)	0.0076 (17)
C50	0.022 (2)	0.0152 (18)	0.0094 (16)	0.0088 (16)	0.0034 (14)	0.0020 (14)
C51	0.017 (2)	0.027 (2)	0.0186 (18)	0.0073 (18)	0.0017 (15)	0.0061 (16)
C52	0.020 (2)	0.034 (2)	0.025 (2)	0.0045 (19)	0.0072 (16)	0.0069 (18)
C53	0.036 (2)	0.024 (2)	0.023 (2)	0.0069 (19)	0.0123 (18)	0.0087 (17)
C54	0.033 (2)	0.024 (2)	0.0194 (19)	0.0145 (19)	0.0069 (16)	0.0070 (16)
C55	0.0203 (19)	0.0184 (19)	0.0160 (17)	0.0104 (16)	0.0049 (14)	0.0034 (15)
C60	0.024 (2)	0.021 (2)	0.0197 (18)	0.0079 (17)	0.0088 (15)	0.0161 (16)
C61	0.023 (2)	0.025 (2)	0.0204 (18)	0.0108 (17)	0.0091 (15)	0.0146 (16)
C62	0.031 (2)	0.045 (3)	0.039 (2)	0.023 (2)	0.0206 (19)	0.029 (2)
C63	0.017 (2)	0.053 (3)	0.046 (3)	0.008 (2)	0.0102 (19)	0.033 (2)
C64	0.025 (2)	0.035 (3)	0.034 (2)	−0.003 (2)	0.0021 (18)	0.019 (2)
C65	0.030 (2)	0.018 (2)	0.024 (2)	0.0051 (18)	0.0097 (17)	0.0119 (16)
C1W	0.039 (3)	0.036 (3)	0.034 (2)	0.021 (2)	0.0078 (19)	0.015 (2)
C3W	0.040 (3)	0.051 (3)	0.039 (3)	0.032 (2)	0.011 (2)	0.014 (2)
C2W	0.049 (3)	0.037 (3)	0.050 (3)	0.023 (3)	0.015 (2)	0.014 (2)

### Geometric parameters (Å, °)

**Table:**

W1—C41	2.000 (4)	N24—C25	1.493 (4)
W1—C44	2.035 (4)	N24—C23	1.506 (4)
W1—C43	2.042 (4)	C12—C13	1.500 (5)
W1—C42	2.046 (4)	C12—H12A	0.9900
W1—C45	2.050 (4)	C12—H12B	0.9900
W2—C35'	1.947 (10)	C13—H13A	0.9900
W2—C31	2.037 (4)	C13—H13B	0.9900
W2—C35	2.038 (10)	C15—C16	1.524 (5)
W2—C33	2.042 (4)	C15—H15A	0.9900
W2—C34	2.049 (4)	C15—H15B	0.9900
W2—C32	2.054 (5)	C16—H16A	0.9900
W3—C3W^i^	2.047 (4)	C16—H16B	0.9900
W3—C3W	2.047 (4)	C22—C23	1.494 (5)
W3—C1W^i^	2.059 (5)	C22—H22A	0.9900
W3—C1W	2.059 (5)	C22—H22B	0.9900
W3—C2W	2.064 (5)	C23—H23A	0.9900
W3—C2W^i^	2.064 (5)	C23—H23B	0.9900
Sn1—W1	2.7274 (3)	C25—C26	1.535 (5)
Sn1—O11	2.091 (2)	C25—H25A	0.9900
Sn1—O17	2.001 (3)	C25—H25B	0.9900
Sn1—O21	2.201 (2)	C26—H26A	0.9900
Sn1—N14	2.507 (3)	C26—H26B	0.9900
Sn2—W2	2.7334 (3)	C35—O35	1.134 (11)
Sn2—O11	2.173 (2)	C35'—O35'	1.181 (11)
Sn2—O21	2.104 (2)	C50—C55	1.381 (5)
Sn2—O27	2.011 (2)	C50—C51	1.393 (5)
Sn2—N24	2.391 (3)	C51—C52	1.387 (5)
O11—C12	1.430 (3)	C51—H51A	0.9500
O17—C16	1.416 (4)	C52—C53	1.383 (6)
O21—C22	1.443 (4)	C52—H52A	0.9500
O27—C26	1.405 (4)	C53—C54	1.384 (5)
O31—C31	1.146 (4)	C53—H53A	0.9500
O32—C32	1.138 (5)	C54—C55	1.399 (5)
O33—C33	1.142 (5)	C54—H54A	0.9500
O34—C34	1.138 (4)	C55—H55A	0.9500
O41—C41	1.146 (4)	C60—C65	1.387 (5)
O42—C42	1.139 (4)	C60—C61	1.389 (5)
O43—C43	1.145 (4)	C61—C62	1.401 (5)
O44—C44	1.152 (4)	C61—H61A	0.9500
O45—C45	1.139 (4)	C62—C63	1.367 (6)
O1W—C1W	1.129 (5)	C62—H62A	0.9500
O2W—C2W	1.117 (5)	C63—C64	1.378 (6)
O3W—C3W	1.140 (5)	C63—H63A	0.9500
N14—C60	1.448 (4)	C64—C65	1.391 (6)
N14—C15	1.498 (5)	C64—H64A	0.9500
N14—C13	1.513 (4)	C65—H65A	0.9500
N24—C50	1.467 (4)		
			
C41—W1—C44	92.39 (14)	C13—C12—H12B	110.1
C41—W1—C43	91.82 (14)	H12A—C12—H12B	108.4
C44—W1—C43	174.81 (14)	C12—C13—N14	109.1 (3)
C41—W1—C42	88.78 (15)	C12—C13—H13A	109.9
C44—W1—C42	91.70 (14)	N14—C13—H13A	109.9
C43—W1—C42	91.42 (14)	C12—C13—H13B	109.9
C41—W1—C45	89.43 (15)	N14—C13—H13B	109.9
C44—W1—C45	86.05 (14)	H13A—C13—H13B	108.3
C43—W1—C45	90.96 (14)	N14—C15—C16	110.1 (3)
C42—W1—C45	177.07 (16)	N14—C15—H15A	109.6
C41—W1—Sn1	176.98 (10)	C16—C15—H15A	109.6
C44—W1—Sn1	88.91 (10)	N14—C15—H15B	109.6
C43—W1—Sn1	86.74 (10)	C16—C15—H15B	109.6
C42—W1—Sn1	93.90 (11)	H15A—C15—H15B	108.2
C45—W1—Sn1	87.94 (11)	O17—C16—C15	111.6 (3)
C35'—W2—C31	98.6 (3)	O17—C16—H16A	109.3
C35'—W2—C35	13.6 (3)	C15—C16—H16A	109.3
C31—W2—C35	90.4 (3)	O17—C16—H16B	109.3
C35'—W2—C33	95.2 (3)	C15—C16—H16B	109.3
C31—W2—C33	87.69 (15)	H16A—C16—H16B	108.0
C35—W2—C33	84.0 (3)	O21—C22—C23	110.2 (3)
C35'—W2—C34	87.6 (3)	O21—C22—H22A	109.6
C31—W2—C34	173.43 (15)	C23—C22—H22A	109.6
C35—W2—C34	95.2 (3)	O21—C22—H22B	109.6
C33—W2—C34	89.53 (15)	C23—C22—H22B	109.6
C35'—W2—C32	86.6 (3)	H22A—C22—H22B	108.1
C31—W2—C32	89.70 (15)	C22—C23—N24	111.3 (3)
C35—W2—C32	97.5 (3)	C22—C23—H23A	109.4
C33—W2—C32	177.00 (15)	N24—C23—H23A	109.4
C34—W2—C32	92.91 (16)	C22—C23—H23B	109.4
C35'—W2—Sn2	170.6 (2)	N24—C23—H23B	109.4
C31—W2—Sn2	90.23 (11)	H23A—C23—H23B	108.0
C35—W2—Sn2	172.2 (2)	N24—C25—C26	110.1 (3)
C33—W2—Sn2	88.23 (11)	N24—C25—H25A	109.6
C34—W2—Sn2	83.74 (11)	C26—C25—H25A	109.6
C32—W2—Sn2	90.29 (12)	N24—C25—H25B	109.6
C3W^i^—W3—C3W	180.0 (3)	C26—C25—H25B	109.6
C3W^i^—W3—C1W^i^	87.14 (16)	H25A—C25—H25B	108.2
C3W—W3—C1W^i^	92.86 (16)	O27—C26—C25	113.9 (3)
C3W^i^—W3—C1W	92.86 (16)	O27—C26—H26A	108.8
C3W—W3—C1W	87.14 (16)	C25—C26—H26A	108.8
C1W^i^—W3—C1W	180.0 (3)	O27—C26—H26B	108.8
C3W^i^—W3—C2W	89.36 (18)	C25—C26—H26B	108.8
C3W—W3—C2W	90.64 (18)	H26A—C26—H26B	107.7
C1W^i^—W3—C2W	88.71 (19)	O31—C31—W2	177.5 (3)
C1W—W3—C2W	91.29 (19)	O32—C32—W2	179.3 (4)
C3W^i^—W3—C2W^i^	90.64 (18)	O33—C33—W2	179.4 (4)
C3W—W3—C2W^i^	89.36 (18)	O34—C34—W2	177.3 (3)
C1W^i^—W3—C2W^i^	91.29 (19)	O35—C35—W2	177.9 (8)
C1W—W3—C2W^i^	88.71 (19)	O35'—C35'—W2	176.3 (8)
C2W—W3—C2W^i^	180.0 (3)	O41—C41—W1	178.9 (3)
O17—Sn1—O11	110.71 (10)	O42—C42—W1	177.8 (4)
O17—Sn1—O21	88.04 (10)	O43—C43—W1	178.3 (3)
O11—Sn1—O21	69.82 (8)	O44—C44—W1	177.1 (3)
O17—Sn1—N14	76.52 (9)	O45—C45—W1	176.8 (4)
O11—Sn1—N14	73.42 (9)	C55—C50—C51	119.6 (3)
O21—Sn1—N14	131.57 (8)	C55—C50—N24	118.8 (3)
O17—Sn1—W1	132.90 (7)	C51—C50—N24	121.6 (3)
O11—Sn1—W1	116.19 (7)	C52—C51—C50	119.6 (4)
O21—Sn1—W1	103.68 (6)	C52—C51—H51A	120.2
N14—Sn1—W1	120.53 (7)	C50—C51—H51A	120.2
O27—Sn2—O21	104.24 (10)	C53—C52—C51	120.9 (4)
O27—Sn2—O11	85.86 (9)	C53—C52—H52A	119.5
O21—Sn2—O11	70.16 (8)	C51—C52—H52A	119.5
O27—Sn2—N24	78.41 (9)	C52—C53—C54	119.6 (4)
O21—Sn2—N24	75.71 (9)	C52—C53—H53A	120.2
O11—Sn2—N24	137.35 (9)	C54—C53—H53A	120.2
O27—Sn2—W2	122.15 (7)	C53—C54—C55	119.7 (4)
O21—Sn2—W2	132.75 (7)	C53—C54—H54A	120.2
O11—Sn2—W2	102.58 (6)	C55—C54—H54A	120.2
N24—Sn2—W2	119.42 (7)	C50—C55—C54	120.6 (3)
C12—O11—Sn1	119.87 (19)	C50—C55—H55A	119.7
C12—O11—Sn2	125.65 (19)	C54—C55—H55A	119.7
Sn1—O11—Sn2	110.16 (9)	C65—C60—C61	118.7 (3)
C16—O17—Sn1	119.0 (2)	C65—C60—N14	118.3 (3)
C22—O21—Sn2	119.90 (18)	C61—C60—N14	122.8 (3)
C22—O21—Sn1	122.88 (19)	C60—C61—C62	119.9 (4)
Sn2—O21—Sn1	108.63 (9)	C60—C61—H61A	120.0
C26—O27—Sn2	118.74 (19)	C62—C61—H61A	120.0
C60—N14—C15	114.3 (3)	C63—C62—C61	121.0 (4)
C60—N14—C13	114.8 (3)	C63—C62—H62A	119.5
C15—N14—C13	110.5 (3)	C61—C62—H62A	119.5
C60—N14—Sn1	109.40 (19)	C62—C63—C64	119.2 (4)
C15—N14—Sn1	102.40 (19)	C62—C63—H63A	120.4
C13—N14—Sn1	104.16 (18)	C64—C63—H63A	120.4
C50—N24—C25	113.4 (3)	C63—C64—C65	120.7 (4)
C50—N24—C23	111.8 (2)	C63—C64—H64A	119.7
C25—N24—C23	111.7 (3)	C65—C64—H64A	119.7
C50—N24—Sn2	114.0 (2)	C60—C65—C64	120.4 (4)
C25—N24—Sn2	100.56 (19)	C60—C65—H65A	119.8
C23—N24—Sn2	104.57 (19)	C64—C65—H65A	119.8
O11—C12—C13	108.0 (3)	O1W—C1W—W3	177.5 (4)
O11—C12—H12A	110.1	O3W—C3W—W3	179.1 (4)
C13—C12—H12A	110.1	O2W—C2W—W3	179.2 (4)
O11—C12—H12B	110.1		
			
C41—W1—Sn1—O17	−110.4 (19)	W1—Sn1—O21—Sn2	121.94 (8)
C44—W1—Sn1—O17	134.13 (14)	O21—Sn2—O27—C26	80.3 (3)
C43—W1—Sn1—O17	−48.71 (14)	O11—Sn2—O27—C26	148.6 (3)
C42—W1—Sn1—O17	42.50 (14)	N24—Sn2—O27—C26	8.4 (2)
C45—W1—Sn1—O17	−139.78 (14)	W2—Sn2—O27—C26	−109.2 (2)
C41—W1—Sn1—O11	63.9 (19)	O17—Sn1—N14—C60	−132.3 (2)
C44—W1—Sn1—O11	−51.63 (12)	O11—Sn1—N14—C60	110.9 (2)
C43—W1—Sn1—O11	125.54 (12)	O21—Sn1—N14—C60	152.7 (2)
C42—W1—Sn1—O11	−143.25 (12)	W1—Sn1—N14—C60	−0.2 (2)
C45—W1—Sn1—O11	34.46 (12)	O17—Sn1—N14—C15	−10.7 (2)
C41—W1—Sn1—O21	−10.1 (19)	O11—Sn1—N14—C15	−127.5 (2)
C44—W1—Sn1—O21	−125.61 (12)	O21—Sn1—N14—C15	−85.7 (2)
C43—W1—Sn1—O21	51.55 (11)	W1—Sn1—N14—C15	121.5 (2)
C42—W1—Sn1—O21	142.76 (12)	O17—Sn1—N14—C13	104.4 (2)
C45—W1—Sn1—O21	−39.52 (12)	O11—Sn1—N14—C13	−12.4 (2)
C41—W1—Sn1—N14	149.3 (19)	O21—Sn1—N14—C13	29.5 (3)
C44—W1—Sn1—N14	33.84 (13)	W1—Sn1—N14—C13	−123.40 (19)
C43—W1—Sn1—N14	−149.00 (13)	O27—Sn2—N24—C50	−150.5 (2)
C42—W1—Sn1—N14	−57.79 (13)	O21—Sn2—N24—C50	101.4 (2)
C45—W1—Sn1—N14	119.93 (12)	O11—Sn2—N24—C50	138.88 (19)
C35'—W2—Sn2—O27	5.0 (16)	W2—Sn2—N24—C50	−29.9 (2)
C31—W2—Sn2—O27	165.61 (13)	O27—Sn2—N24—C25	−28.8 (2)
C35—W2—Sn2—O27	−99.6 (18)	O21—Sn2—N24—C25	−137.0 (2)
C33—W2—Sn2—O27	−106.71 (13)	O11—Sn2—N24—C25	−99.5 (2)
C34—W2—Sn2—O27	−16.99 (14)	W2—Sn2—N24—C25	91.7 (2)
C32—W2—Sn2—O27	75.91 (13)	O27—Sn2—N24—C23	87.1 (2)
C35'—W2—Sn2—O21	172.6 (16)	O21—Sn2—N24—C23	−21.01 (19)
C31—W2—Sn2—O21	−26.87 (13)	O11—Sn2—N24—C23	16.5 (2)
C35—W2—Sn2—O21	67.9 (18)	W2—Sn2—N24—C23	−152.35 (17)
C33—W2—Sn2—O21	60.82 (13)	Sn1—O11—C12—C13	47.2 (3)
C34—W2—Sn2—O21	150.54 (14)	Sn2—O11—C12—C13	−107.1 (3)
C32—W2—Sn2—O21	−116.57 (13)	O11—C12—C13—N14	−56.1 (3)
C35'—W2—Sn2—O11	98.1 (16)	C60—N14—C13—C12	−80.8 (3)
C31—W2—Sn2—O11	−101.36 (12)	C15—N14—C13—C12	148.2 (3)
C35—W2—Sn2—O11	−6.6 (18)	Sn1—N14—C13—C12	38.9 (3)
C33—W2—Sn2—O11	−13.68 (12)	C60—N14—C15—C16	151.8 (3)
C34—W2—Sn2—O11	76.04 (13)	C13—N14—C15—C16	−76.8 (3)
C32—W2—Sn2—O11	168.94 (12)	Sn1—N14—C15—C16	33.6 (3)
C35'—W2—Sn2—N24	−89.7 (16)	Sn1—O17—C16—C15	43.3 (4)
C31—W2—Sn2—N24	70.91 (13)	N14—C15—C16—O17	−51.5 (4)
C35—W2—Sn2—N24	165.7 (18)	Sn2—O21—C22—C23	27.6 (4)
C33—W2—Sn2—N24	158.59 (13)	Sn1—O21—C22—C23	−116.6 (3)
C34—W2—Sn2—N24	−111.69 (13)	O21—C22—C23—N24	−47.2 (4)
C32—W2—Sn2—N24	−18.79 (13)	C50—N24—C23—C22	−81.7 (3)
O17—Sn1—O11—C12	−86.7 (2)	C25—N24—C23—C22	150.0 (3)
O21—Sn1—O11—C12	−166.5 (3)	Sn2—N24—C23—C22	42.1 (3)
N14—Sn1—O11—C12	−18.6 (2)	C50—N24—C25—C26	165.6 (3)
W1—Sn1—O11—C12	97.8 (2)	C23—N24—C25—C26	−67.0 (4)
O17—Sn1—O11—Sn2	71.24 (12)	Sn2—N24—C25—C26	43.5 (3)
O21—Sn1—O11—Sn2	−8.51 (10)	Sn2—O27—C26—C25	14.6 (4)
N14—Sn1—O11—Sn2	139.37 (13)	N24—C25—C26—O27	−42.8 (4)
W1—Sn1—O11—Sn2	−104.25 (9)	C25—N24—C50—C55	−152.8 (3)
O27—Sn2—O11—C12	58.5 (3)	C23—N24—C50—C55	79.9 (4)
O21—Sn2—O11—C12	165.3 (3)	Sn2—N24—C50—C55	−38.5 (3)
N24—Sn2—O11—C12	126.4 (2)	C25—N24—C50—C51	28.4 (4)
W2—Sn2—O11—C12	−63.5 (3)	C23—N24—C50—C51	−99.0 (4)
O27—Sn2—O11—Sn1	−97.87 (12)	Sn2—N24—C50—C51	142.7 (3)
O21—Sn2—O11—Sn1	8.89 (10)	C55—C50—C51—C52	0.8 (5)
N24—Sn2—O11—Sn1	−29.95 (18)	N24—C50—C51—C52	179.6 (3)
W2—Sn2—O11—Sn1	140.09 (9)	C50—C51—C52—C53	−0.8 (6)
O11—Sn1—O17—C16	49.0 (3)	C51—C52—C53—C54	0.6 (6)
O21—Sn1—O17—C16	116.6 (3)	C52—C53—C54—C55	−0.5 (6)
N14—Sn1—O17—C16	−17.1 (3)	C51—C50—C55—C54	−0.7 (5)
W1—Sn1—O17—C16	−136.5 (2)	N24—C50—C55—C54	−179.5 (3)
O27—Sn2—O21—C22	−76.9 (2)	C53—C54—C55—C50	0.5 (5)
O11—Sn2—O21—C22	−157.1 (3)	C15—N14—C60—C65	−56.2 (4)
N24—Sn2—O21—C22	−3.1 (2)	C13—N14—C60—C65	174.6 (3)
W2—Sn2—O21—C22	114.0 (2)	Sn1—N14—C60—C65	58.0 (3)
O27—Sn2—O21—Sn1	71.81 (12)	C15—N14—C60—C61	127.9 (3)
O11—Sn2—O21—Sn1	−8.36 (10)	C13—N14—C60—C61	−1.3 (5)
N24—Sn2—O21—Sn1	145.64 (12)	Sn1—N14—C60—C61	−117.9 (3)
W2—Sn2—O21—Sn1	−97.32 (10)	C65—C60—C61—C62	1.3 (5)
O17—Sn1—O21—C22	43.4 (2)	N14—C60—C61—C62	177.2 (3)
O11—Sn1—O21—C22	156.3 (3)	C60—C61—C62—C63	−1.7 (6)
N14—Sn1—O21—C22	113.4 (2)	C61—C62—C63—C64	0.6 (6)
W1—Sn1—O21—C22	−90.5 (2)	C62—C63—C64—C65	0.9 (6)
O17—Sn1—O21—Sn2	−104.22 (11)	C61—C60—C65—C64	0.2 (5)
O11—Sn1—O21—Sn2	8.71 (10)	N14—C60—C65—C64	−175.9 (3)
N14—Sn1—O21—Sn2	−34.23 (17)	C63—C64—C65—C60	−1.3 (6)

Symmetry codes: (i) −*x*, −*y*, −*z*.

### Hydrogen-bond geometry (Å, °)

**Table:**

*D*—H···*A*	*D*—H	H···*A*	*D*···*A*	*D*—H···*A*
C23—H23a···O44^ii^	0.99	2.52	3.301 (5)	136
C64—H64a···O31^iii^	0.95	2.58	3.174 (6)	121

Symmetry codes: (ii) *x*+1, *y*, *z*; (iii) *x*−1, *y*−1, *z*.
